# Final farewell to Claudio Rapezzi: observation, deduction and knowledge in medicine

**DOI:** 10.3389/fcvm.2023.1221983

**Published:** 2023-06-12

**Authors:** Aldostefano Porcari, Gianfranco Sinagra, Cristina Candida Quarta, Marianna Fontana, Julian D. Gillmore

**Affiliations:** ^1^ National Amyloidosis Centre, Division of Medicine, University College London, London, United Kingdom; ^2^ Centre for Diagnosis and Treatment of Cardiomyopathies, Cardiovascular Department, Azienda Sanitaria Universitaria Giuliano-Isontina (ASUGI), University of Trieste, European Reference Network for Rare, Low Prevalence and Complex Diseases of the Heart-ERN GUARD-Heart, Trieste, Italy; ^3^ European Reference Network for Rare, Low Prevalence and Complex Diseases of the Heart-ERN GUARD-Heart

**Keywords:** amyloidosis, diagnosis, cardiology, in memoriam, mentor

Many colleagues have known him as a brilliant mind and prominent member of the cardiological and amyloidosis community, many others as a trustful and distinguished partner in research with flashes of extraordinary intelligence and an exceptional ability for lateral thinking, and many more as a close friend with extremely sharp irony and culture. However, Professor Claudio Rapezzi was primarily a mentor with the unique ability to ignite the minds and the hearts of young researchers and colleagues with his scientific passion.

**Figure 1:**
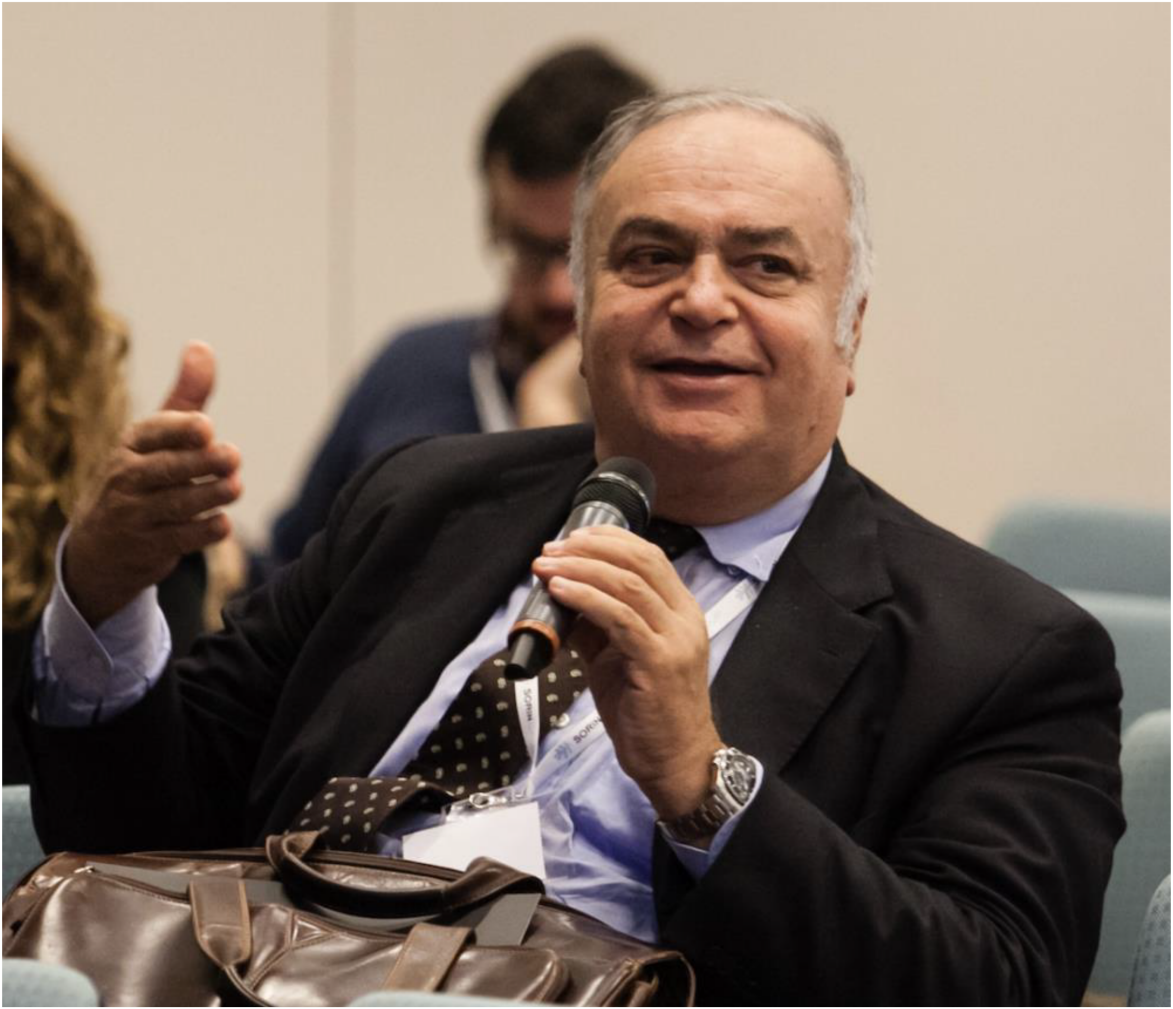
Professor Claudio Rapezzi during a discussion at the “Advances in Heart Failure, Cardiomyopathies and Pericardial Diseases” held in Trieste (Italy).

Although he was aware of his exceptional qualities, Prof. Rapezzi was extremely humble, he loved spending time in his small office writing of science, and despite being extremely busy, his door was always open to everyone. He had a natural disposition in human relationships, promoting a safe environment for young physician to improve their medical knowledge. He was a progressive thinker and, as a teacher, he embodied the qualities of curiosity, critical thinking, observation, passion and creativity (Figure 1). Meeting Professor Rapezzi marked a fundamental moment in the career of many young researchers. The path of those who had the privilege to be supervised by or to collaborate with him was influenced in many different and sometimes unexpected ways.

William Osler once said “*The whole art of medicine is in observation… but to educate the eye to see, the ear to hear and the finger to feel takes time, and to make a beginning, to start a man on the right path, is all that you can do*”. Professor Claudio Rapezzi dedicated his life to the study of medicine and became a master in the art of observation. During a career spanning almost 50 years, he has deeply transformed the cardiological and the amyloidosis community worldwide.

With his unique qualities of curiosity, imagination and immense culture, he was the exemplar of the physician and detective character with his ability to spot inconsistencies in challenging clinical scenarios in a contemporary version of Sherlock Holmes (1). He shared with the cardiology community the “red flag approach” in cardiomyopathies and, particularly, in amyloidosis (2, 3). Indeed, one of the mottos he loved and was used to share was “When you have eliminated the impossible, whatever remains, however improbable, must be the truth” (The Sign of the Four, 1890), and this is exactly how he was often teaching his students and peers how to approach the suspicion and identification of rare cardiac diseases.

He was among the first researchers to understand the key value of carpal tunnel syndrome as an early clinical marker of future development of cardiac amyloidosis (4, 5).

Professor Rapezzi considered electrocardiography as the longest-running tool for non-invasive tissue characterisation. Through his expert interpretation, Professor Rapezzi was able to explore the presence of amyloid deposition in the heart and to predict what endomyocardial biopsy and cardiac magnetic resonance would have demonstrated many years later (6, 7). Professor Rapezzi was very passionate on dissecting the heterogeneous clinical phenotype of ATTR amyloidosis (8, 9). His classification of ATTR clinical phenotypes (cardiac, neurological and mixed) has been implemented worldwide by clinicians, researchers and pharmaceutical companies. He coordinated the Transthyretin Amyloid Outcome Survey (THAOS), with the final aim of understanding and characterizing the natural history of ATTR amyloidosis (10). In early 2000s, Professor Claudio Rapezzi was a pioneer in investigating the clinical use of scintigraphy with bone tracers for the diagnosis of cardiac amyloidosis (11, 12). Ten years later, that intuition paved the way for the development of a non-invasive algorithm for the diagnosis of transthyretin cardiac amyloidosis in an international collaboration with the National Amyloidosis Centre (London, UK) which has substantially changed the paradigm for diagnosing ATTR cardiac amyloidosis and has dramatically reduced the need for invasive endomyocardial biopsy (13–15). Ideally, every diagnosis made without the need for cardiac biopsy is a tribute to the fine intelligence and original intuition of Professor Claudio Rapezzi and those who collaborated with him over time.

**Figure 2:**
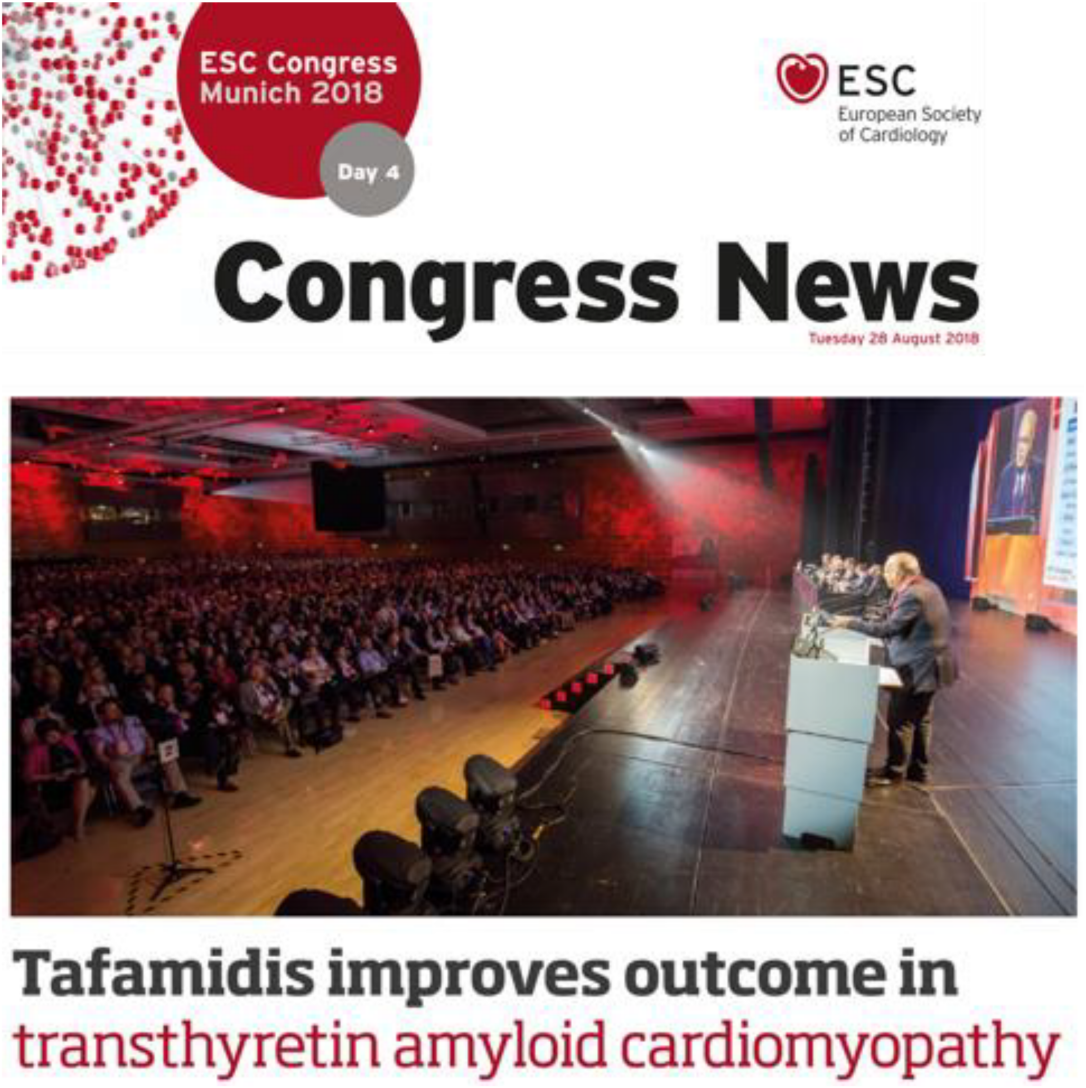
Professor Rapezzi presenting the results of the ATTR-ACT study at the European society of cardiology congress held in Munich.

Professor Rapezzi coordinated the international phase 3 Safety and Efficacy of Tafamidis in Patients With Transthyretin Cardiomyopathy (ATTR-ACT) trial of tafamidis (16), which is the only drug ever tested in ATTR cardiac amyloidosis to have impacted survival. In 2018, he presented the results of the ATTR-ACT at the European Society of Cardiology Congress held in Munich and ignited the audience with his passion and culture (Figure 2). Professor Rapezzi considered tafamidis as a drug of “firsts”, highlighting four themes:

• The first drug to show efficacy in ATTR cardiac amyloidosis;
• The first example of precision medicine in the treatment of a cardiomyopathy;
• The first drug to show efficacy in heart failure with preserved ejection fraction;
• The first drug without anti-neurohormonal activity to show efficacy in heart failure;

The ATTR-ACT study has transformed the treatment of ATTR cardiac amyloidosis and revolutionised the lives of patients suffering from this increasingly diagnosed cause of heart failure; in no small way, this is thanks to Professor Rapezzi.

On top of his undisputed scientific expertise, Professor Rapezzi was highly considered for his critical approach to the methodology of advancing medical knowledge. The question of “nosology” and its application in cardiomyopathies was central in Professor Rapezzi's vision, who was a great estimator of the writer Umberto Eco and the philosopher Karl Popper. A recent stimulating example is represented by the impossible interview between Sherlock Holmes and David Sackett about the fundamental question “how much can we trust the guidelines?” (17). Professor Rapezzi's pupils will never forget his positive approach to “error in medicine” as a source of thinking and a unique opportunity for advancing medical understanding. In this field, Professor Rapezzi coordinated an international group of researchers with the aim of defining criteria for classification of cardiomyopathies in 2008 (18). Understanding that the boundaries of restrictive cardiomyopathy have become increasingly blurred, he recently identified the limitations of the classification of restrictive cardiomyopathy encountered in clinical practice today and proposed a new definition of this specific form of heart disease (19).

Professor Rapezzi dedicated the last years of his career to the foundation of the Italian Network for Cardiac Amyloidosis (20), with the aim of promoting collaboration among Italian centres involved in the care of patients with suspected or confirmed cardiac amyloidosis. The legacy of Professor Rapezzi continues today with the many ideas and research questions that awaits to be explored by his pupils disseminated worldwide: the role of electrocardiography in the contemporary care of patients with cardiac amyloidosis (21), the cardiomyopathy-oriented interpretation of echocardiographic findings (22, 23), the association between cardiac amyloidosis and aortic stenosis, the many questions on the mechanisms of bone tracers binding to the amyloid infiltrated heart (24), the clinical usefulness of tafamidis in ATTR-CM patients with NYHA functional class III symptoms, the correlation between genotype and phenotype in ATTR amyloidosis (25), gender differences in ATTR-CM (26–28), the new frontiers in treatment strategies and the possibility of combination therapy (29).

Professor Claudio Rapezzi was a giant of the amyloid field. It is worth asking to ourselves “What the amyloidosis culture in Italy and in the world would have been without Professor Rapezzi?”. How will the field keep progressing without him?”. Most of his former collaborators and peers were actually wondering what innovations his brilliant mind was conceiving when he showed up at the 2022 International Society of Amyloidosis meeting in Heidelberg.

We will never forget his legendary humour and immense culture during his scientific talks or chairing sessions in the countless meetings across the world.

From his legacy, we should all start approaching the medical field by dissecting the overall picture into its multiple and detailed features, like he was used to do when he referred to the Pink Floyd Prism album cover when talking about the different aspects and heterogeneity of amyloidosis.

We are close to his beloved Marinella, friends and colleagues in Ferrara, Bologna and all around the world.

He will be greatly missed and will continue being an inspiration for the next generations.
